# Dynamic regulation of coral energy metabolism throughout the diel cycle

**DOI:** 10.1038/s41598-020-76828-2

**Published:** 2020-11-16

**Authors:** Lauren Buckley Linsmayer, Dimitri Dominique Deheyn, Lars Tomanek, Martin Tresguerres

**Affiliations:** 1 Scripps Institution of Oceanography, University of California San Diego, La Jolla, CA 92093 USA; 2 Department of Biological Sciences, California Polytechnic State University, San Luis Obispo, CA 93407-0401 USA

**Keywords:** Marine biology, Oxidoreductases, Homeostasis, Animal physiology

## Abstract

Coral reefs are naturally exposed to daily and seasonal variations in environmental oxygen levels, which can be exacerbated in intensity and duration by anthropogenic activities. However, coral’s diel oxygen dynamics and fermentative pathways remain poorly understood. Here, continuous oxygen microelectrode recordings in the coral diffusive boundary layer revealed hyperoxia during daytime and hypoxia at nighttime resulting from net photosynthesis and net respiration, respectively. The activities of the metabolic enzymes citrate synthase (CS), malate dehydrogenase, and strombine dehydrogenase remained constant throughout the day/night cycle, suggesting that energy metabolism was regulated through adjustments in metabolite fluxes and not through changes in enzyme abundance. Liquid chromatography-mass spectrometry analyses identified strombine as coral’s main fermentative end product. Strombine levels peaked as oxygen became depleted at dusk, indicating increased fermentation rates at the onset of nightly hypoxia, and again at dawn as photosynthesis restored oxygen and photosynthate supply. When these peaks were excluded from the analyses, average strombine levels during the day were nearly double those at night, indicating sifnificant fermentation rates even during aerobic conditions. These results highlight the dynamic changes in oxygen levels in the coral diffusive boundary layer, and the importance of fermentative metabolism for coral biology.

## Introduction

Oxygen (O_2_) concentrations in the bulk seawater overlying coral reefs fluctuate from supersaturation during the day to below saturation at night due to the balance between net photosynthesis and net respiration by the coral holobiont and associated reef organisms^1–3^. Due to the existence of a diffusive boundary layer (DBL) surrounding corals, this microenvironment experiences even more dramatic O_2_ fluctuations than the surrounding seawater^4,5^. High rates of net photosynthesis by corals’ endosymbiotic dinoflagellate algae create hyperoxia in the coral DBL during exposure to light, while net “holobiont” respiration may result in hypoxia or even anoxia throughout exposure to darkness. However, O_2_ dynamics in the coral DBL have most extensively been studied over shorts periods of time, usually just a few minutes^4,5^. To assess the extent to which corals may regularly experience hyperoxia and hypoxia, we performed continuous O_2_ microsensor measurements in the coral DBL throughout an entire day/night (“diel”) cycle.

Because O_2_ is the main terminal electron acceptor in aerobic metabolism, O_2_ availability has critical implications for energy budgets, as it determines the balance between aerobic and anaerobic metabolic pathways, the types of substrates oxidized, the efficiency of energy production, and the degree of intracellular acidification that is induced^6^. Coral O_2_ consumption rates can be up to seven times greater in the light than in the dark, which reflects the stimulatory effect of photosynthetic O_2_ and photosynthate supply on aerobic respiration^5,7^. Conversely, lower O_2_ levels at night would suggest an increased reliance on fermentation. Based on changes in the mRNA levels of some glycolytic and electron transport chain enzymes in corals, many studies have suggested diel adjustments of energy metabolism^8–10^. However, the reported mRNA expression patterns are often inconsistent among the different studies, and evidence linking gene and protein expression is lacking. To better understand potential diel adjustments in the metabolic capacity of corals, we adopted a traditional biochemical approach and measured enzymatic activity of the key metabolic enzymes citrate synthase (CS) and malate dehydrogenase (MDH) at multiple time-points throughout an entire day-night cycle. Additionally, we conducted a global proteomics analysis in an attempt to identify additional metabolic enzymes and putative diel changes in protein abundance.

The continuous anaerobic production of ATP depends on the ability to regenerate NAD^+^ from NADH in the last step of fermentation. In most vertebrates, this reaction is catalyzed by lactate dehydrogenase (LDH) and is coupled to the oxidation of pyruvate into lactate. In contrast, many invertebrates use functionally analogous ‘opine’ dehydrogenases that regenerate NAD^+^ while reductively condensing pyruvate with an amino acid, resulting in an imino acid as the end product. Specifically, octopine dehydrogenase (ODH), strombine dehydrogenase (SDH), and alanopine dehydrogenase (ADH) condense pyruvate with arginine, glycine, and alanine into octopine, strombine, and alanopine, respectively (reviewed in^6,11^). For example, cephalopods use ODH as their terminal dehydrogenase^12^, and intertidal bivalves and worms predominantly use SDH and ADH^13–16^. While different cnidarians have been reported to possess LDH-, ODH-, SDH- and ADH-like activities [reviewed in^11^], the hermatypic coral *Montipora capitata* only demonstrated SDH- and ADH-like activities^17^. However, substrate utilization of opine dehydrogenases is notoriously promiscuous, and thus in vitro enzymatic assays do not necessarily reflect their in vivo function^18–20^. Additionally, substrate specificity and products of opine dehydrogenases cannot be predicted based on amino acid sequence homology^21^, which prevents identifying terminal dehydrogenases using bioinformatic approaches.

In this study, we sought to characterize the main fermentative pathway utilized by corals by measuring the enzymatic activities of terminal dehydrogenases, and by identifying coral fermentative end products using highly sensitive liquid chromatography (LC) and mass spectrometry (MS) methods. Finally, we quantified metabolite abundance at various time points which, combined with the O_2_ microelectrode measurements and the enzymatic assays, allowed us to explore the importance of coral fermentation in relation to [O_2_] in the DBL throughout a complete diel cycle.

## Results and discussion

### O_2_ levels in the coral DBL are highly dynamic

**Figure 1:**
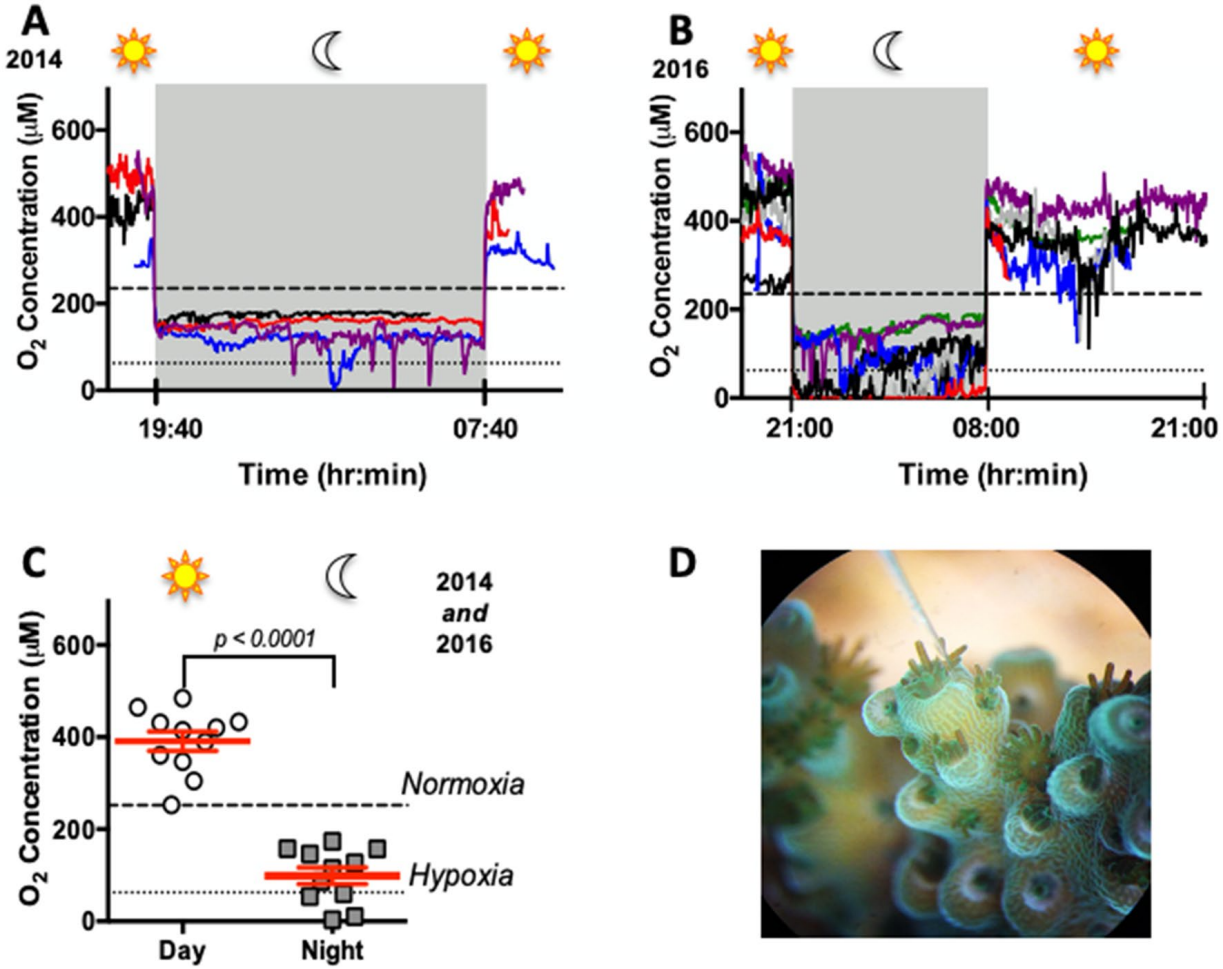
O_2_ concentration in the coral diffusive boundary layer (DBL) throughout a diel cycle. (**A**) Continuous [O_2_] measurements in the DBL of coral branches from the 2014 experiment (n = 4). (**B**) Same as in *A*, but from the 2016 experiment (n = 7). (**C**) Average [O_2_] in the coral DBL during day and night. The white circles and grey squares show the average [O_2_] measurements for each coral branch during day and night, and the red lines represent mean ± SEM. Two-tailed paired *t*-test, n = 11 coral branches measured during 2014 and 2016. The dashed and dotted lines show normoxic [O_2_] in the tank (235 μM) and nominal hypoxia (62.5 μM), respectively. (**D**) Picture of an O_2_ microsensor in the DBL of an *A. yongei* branch taken through a dissecting scope (photo credit: L.B. Linsmayer).

Continuous (> 16 h) O_2_ microsensor measurements were performed in the DBL of 11 *Acropora yongei* branches, four in 2014 (Fig. 1A) and another seven in 2016 (Fig. 1B). As previously reported in short-term experiments^4,5^, the swings in DBL [O_2_] during day-night-day transitions were very rapid and took place within ~ 2 min. However, our longer O_2_ recordings allowed us to characterize DBL [O_2_] during an entire diel cycle. The average DBL [O_2_] throughout the day was 381.3 ± 21.8 μM O_2_; and the average nightly DBL [O_2_] was 73.9 ± 22.9 μM O_2_ (Fig. 1C). Daily hyperoxia resulted from photosynthetic activity by the coral endosymbionts, while average DBL [O_2_] levels at night were within the range of nominal hypoxia (~ 60–90 μM O_2_^22^) and can be attributed to aerobic respiration by coral, algae and other microbes combined with the absence of photosynthetic activity.

To our surprise, there were highly dynamic [O_2_] fluctuations during both day and night (Fig. 1A,B). The DBL [O_2_] variability during both day and night could have been caused by sudden bursts of metabolic activity related to heterotrophic food acquisition or circadian rhythms^23^, as well as by changes in microfluidic dynamics due to ciliary movement^24^. And although the O_2_ electrode was positioned on the coenosarc (Fig. 1D), interference by polyp movement cannot be completely ruled out. During daytime, DBL [O_2_] fluctuations could have additionally been induced by dynamic factors affecting photosynthesis such as photosystem oxidative damage^25^ and photorespiration^5,26^. However, these factors are unlikely to be significant during the relatively mild illumination conditions in our experimental aquarium (~ 200 μmol photons m^−2^ s^−1^). Importantly, the coral branches used in this study were of similar size and shape and were held under homogenous water movement and consistent illumination conditions. In contrast, coral colonies in the wild have variable morphology and are exposed to changing water flow rates and solar intensity as a result of currents, winds, more gradual dark–light-dark transitions at dawn and dusk, shading and clouding. These parameters are bound to result in much more extreme and stochastic changes in the DBL [O_2_] of corals on a natural reef.

### Lack of major changes in coral energy metabolic enzyme activities throughout the diel cycle

**Figure 2:**
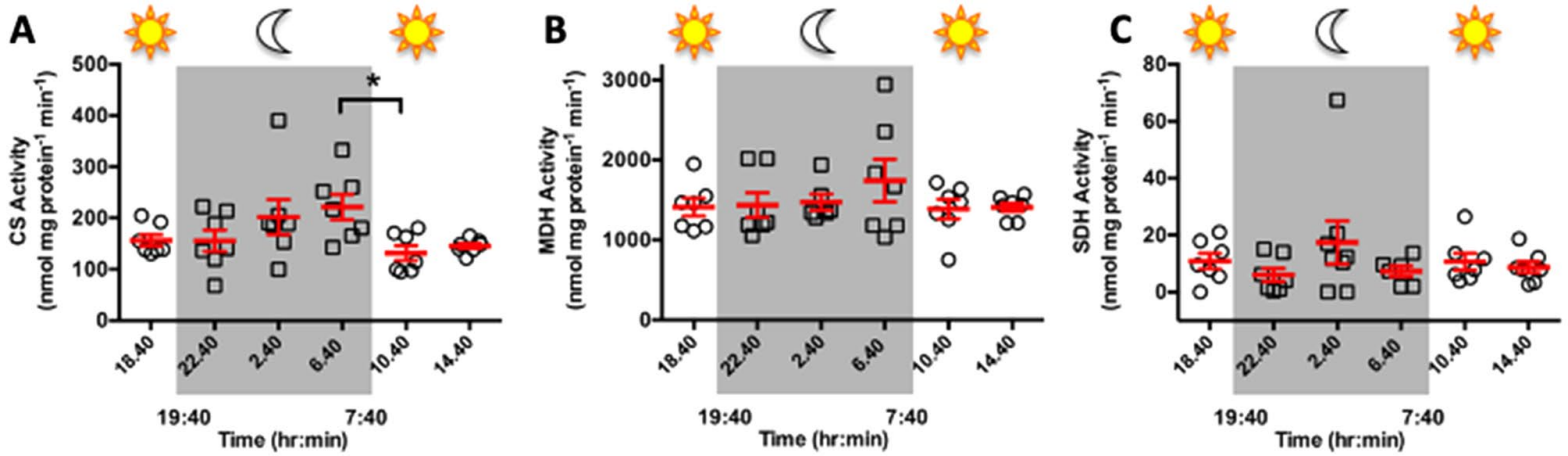
Metabolic enzyme activity throughout a diel cycle. (**A**) Citrate synthase (CS); (**B**) Malate dehydrogenase (MDH); (**C**) Strombine dehydrogenase (SDH) activities (n = 6–8). The only significant difference between timepoints was for CS between 6.40 h and 10.40 h. One-way ANOVA on square root-transformed data (*p* = 0.0265) followed by Tukey’s post-test (which corrects for multiple comparisons) (*p* < 0.05).

The activities of the metabolic enzymes CS, MDH, LDH, ADH, and SDH were measured in *A. yongei* sampled at six timepoints throughput a diel cycle in the 2014 experiment. CS is the rate-limiting enzyme of the TCA cycle and activity measurements are traditionally used as a proxy for aerobic capacity in all animals including photosymbiotic cnidarians^27^. In our coral samples, average CS activity was 168.8 ± 9.4 nmol mg protein^−1^ min^−1^, which is within the range of previous reports for corals (between ~ 2 and 1600 nmol mg protein^−1^ min^−1^)^28–31^. The only statistically significant difference in CS activity was between the last night point (6.40 am) and the first day point (10.40 am) (221.6 ± 24.8 vs. 131.6 ± 14.5 nmol mg protein^−1^ min^−1^) (ANOVA followed by Holm-Tukey test) (Fig. 2A).

MDH is another enzyme of the TCA cycle, and it additionally plays important roles in multiple other metabolic processes including amino acid synthesis, gluconeogenesis, maintenance of redox balance, metabolite exchange between cytoplasm and subcellular compartments, and glyoxylate bypass^32^. In our coral samples, average MDH activity was 1467 ± 61 nmol mg protein^−1^ min^−1^ and remained relatively constant throughout the diel cycle (Fig. 2B).

In the case of terminal dehydrogenases, *A. yongei* did not demonstrate LDH or ADH activities. Furthermore, untargeted metabolomic analyses performed on the same coral samples from this study did not detect putative lactate in *A. yongei*^33^. On the other hand, *A. yongei* tissue extracts did exhibit robust SDH activity with average values of 10.4 ± 1.7 nmol mg protein^-1^ min^-1^. There were no differences in average SDH activity among time points (Fig. 2C). These values are comparable to those reported for *M. capitata*^17^, the only other reef-building coral in which SDH activity has been examined to date.

Putative changes in the abundance of other energy metabolism proteins were explored using 2D polyacrylamide gel electrophoresis (PAGE) followed by matrix-assisted laser desorption/ionization and tandem time-of-flight (MALDI TOF/TOF) mass spectrometry. These analyses identified the oxidative phosphorylation enzymes isocitrate dehydrogenase-NADP^+^ and ATP synthase subunit beta (ATP synthase β), the glycolytic enzymes glyceraldehyde-3-phosphate dehydrogenase (GAPDH; three isoforms) and fructose bisphosphate aldolase (three isoforms), as well as the nitrogen metabolism enzyme glutamine synthase. The only consistent detectable differences were for ATP synthase β, which exhibited statistically significantly higher protein levels at the end of the night. In fact, only a small portion of the proteins (8 out of 62) exhibited significant changes in abundance throughout the diel cycle (Table S1). The multiple potential roles and cellular localization of these proteins, together with the scarce information about coral cell biology^34^, precludes us from making any speculations about the physiological significance of these changes.

In summary, the majority of metabolic enzymes seem to be expressed at relatively constant levels throughout the diel cycle, which reflects a balance between de novo protein synthesis and degradation. In addition, the relative increase in the activity of CS and abundance of ATP synthase β observed at the end of the night might indicate increased protein synthesis over degradation at a time when aerobic respiration is presumably less active. These results are consistent with previously reported complex diel expression patterns of genes involved in energy metabolism. For example, circadian- and hypoxia-dependent regulation results in the cyclical expression of mRNAs coding for glycolytic enzymes, which tend to peak at nighttime^8^.

### Strombine is the main fermentative end-product in corals

**Figure 3:**
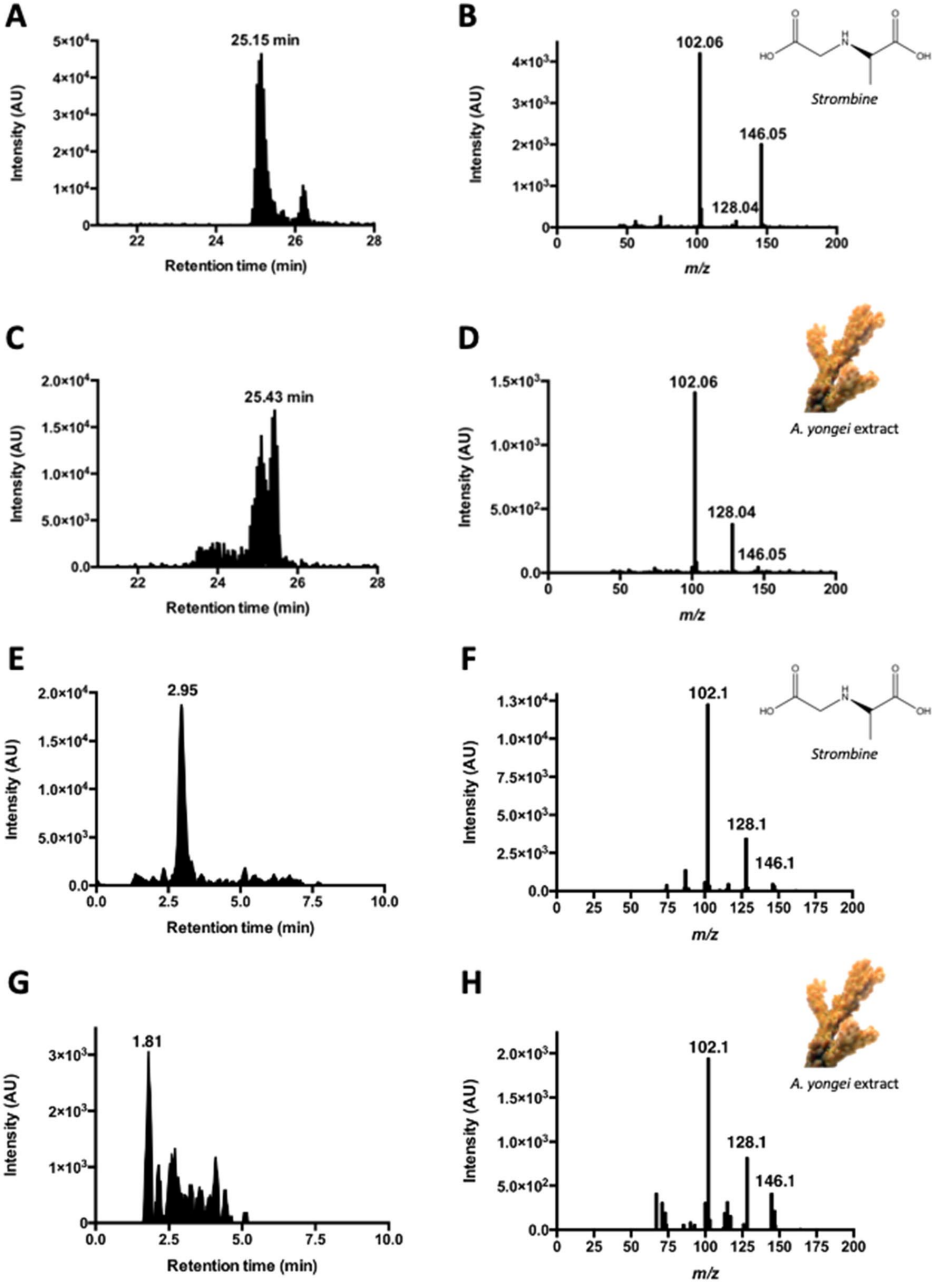
LC–MS analysis of unfiltered and Celite-filtered strombine standard and coral extract. Liquid chromatography extracted ion chromatograms (EICs; left column) and MS/MS spectra (right column) of unfiltered (**A**–**D**) and Celite-filtered (**E**–**H**) pure strombine (**A**,**B**,**E**,**F**) and coral extract (**C**,**D**,**G**,**H**). Retention times are provided for the most intense peaks on the EICs. For both experiments, MS/MS was performed on the parent mass of strombine, *m/z* = 146, and the resulting MS/MS spectra of the extract (**D**,**H**) matches that of the standard (**B**,**F**), with daughter ions at *m/z* 102 and *m/z* 128 and the *m/z* 146 unfragmented parent ion (*A. yongei* photo credit: G.T. Kwan).

Although the enzymatic assays indicated that SDH was the main terminal dehydrogenase in *A. yongei*, in vitro enzymatic assays do not necessarily match the fermentative pathway that is used in vivo, and this especially holds true for opine dehydrogenases^18–20^. Thus, we next used analytical chemistry methods to identify and quantify corals’ fermentative end product(s). Using Multiple Reaction Monitoring (MRM) LC–MS and the mass-to-charge ratios (*m/z*) of authentic opine compounds as reference, *A. yongei* tissues had high levels of a strombine-like compound, much lower levels of an alanopine-like compound, and no octopine or nopaline. Subsequent LC–MS/MS measurements on a QqQ mass spectrometer confirmed the coral strombine-like compound to indeed be strombine (Fig. 3A–D). This technique provides superior specificity for the identification of compounds through the isolation and identification of distinguishing parent and daughter ions, and is considered the “gold standard” of metabolite identification^35^. The LC extracted ion chromatogram (EIC) of the coral strombine compound extracted around a retention time (RT) of 25 min and exhibited a 146.05 *m/z* peak that corresponds to unfragmented strombine (the difference with the exact mass of 147.05 g/mol is due to deprotonation caused by working on negative ionization mode). As a parent ion, coral strombine produced two major daughter ions that matched those derived from the strombine standard. One daughter ion is the result of a dehydration reaction (H_2_O = 18 *m/z*) and appeared at 128.04 *m/z*, while the other daughter ion is produced by decarboxylation (CO_2_ = 44 *m/z*) and appeared at 102.06 *m/z* (Fig. 3B,D).

We further confirmed the identity of this coral compound as strombine using a different LC–MS platform after filtering the coral extract and strombine standard through a column with diatomaceous earth (Celite) commonly used to filter out metal salts (Fig. 3E–H). The difference in peak retention times between the strombine standard (2.95 min) and coral strombine (1.81 min) (Fig. 3E,G) is likely due to ionic interactions with buffers co-extracted in the samples^36^ and to the multiple potential protonation states of opines^13^. Nonetheless, the MS/MS spectra of the coral extract contained the characteristic daughter ions of strombine (*m/z* 128 and *m/z* 102) (Fig. 3F,H). In combination with the enzyme activity assay results (Fig. 1), these LC–MS results demonstrate that corals produce strombine as their main fermentative end-product.

Why do corals produce strombine, and more broadly opines, as their main fermentative end-product? One potential explanation is that the type and amount of opines is typically correlated to the concentration of the corresponding amino acid^19^. For example, the mussel *Mytilus edulis* and the lugworm *Arenicola marina* have high glycine levels and predominantly produce strombine, while the whelk *Littorina littorea* has high alanine levels and produces mostly alanopine^19^. Coral cells generally contain much more glycine than alanine^37,38^, and this matches the robust SDH enzymatic activities (as well as the ~ 80-fold higher strombine levels compared to alanopine that is shown below). The functional significance of producing opines over lactate has been debated for decades. While early studies suggested that opine production induced a less pronounced acidification compared to that of lactate^39^, that hypothesis was subsequently refuted based on theoretical analyses showing equivalent H^+^ yields relative to ATP hydrolysis^16,40^. Other more favored explanations include maintaining a stable osmotic intracellular environment upon glycogen mobilization^14^, and sustaining a lower cytoplasmic NADH/NAD^+^ ratio that enables a higher glycolytic rate^41,42^. Another important consideration is that opines, unlike lactate, are generally retained within the cells rather than being exported. For invertebrate animals without closed circulatory systems such as corals, this may be advantageous because it would prevent the loss of the energy-rich carbon skeleton of opines to the surrounding seawater, which instead could be oxidized for ATP production or reconverted to substrate in situ^14^. In any case, the proposed advantages of opines are not mutually exclusive.

### Dynamic changes in opine abundance throughout day and night

**Figure 4:**
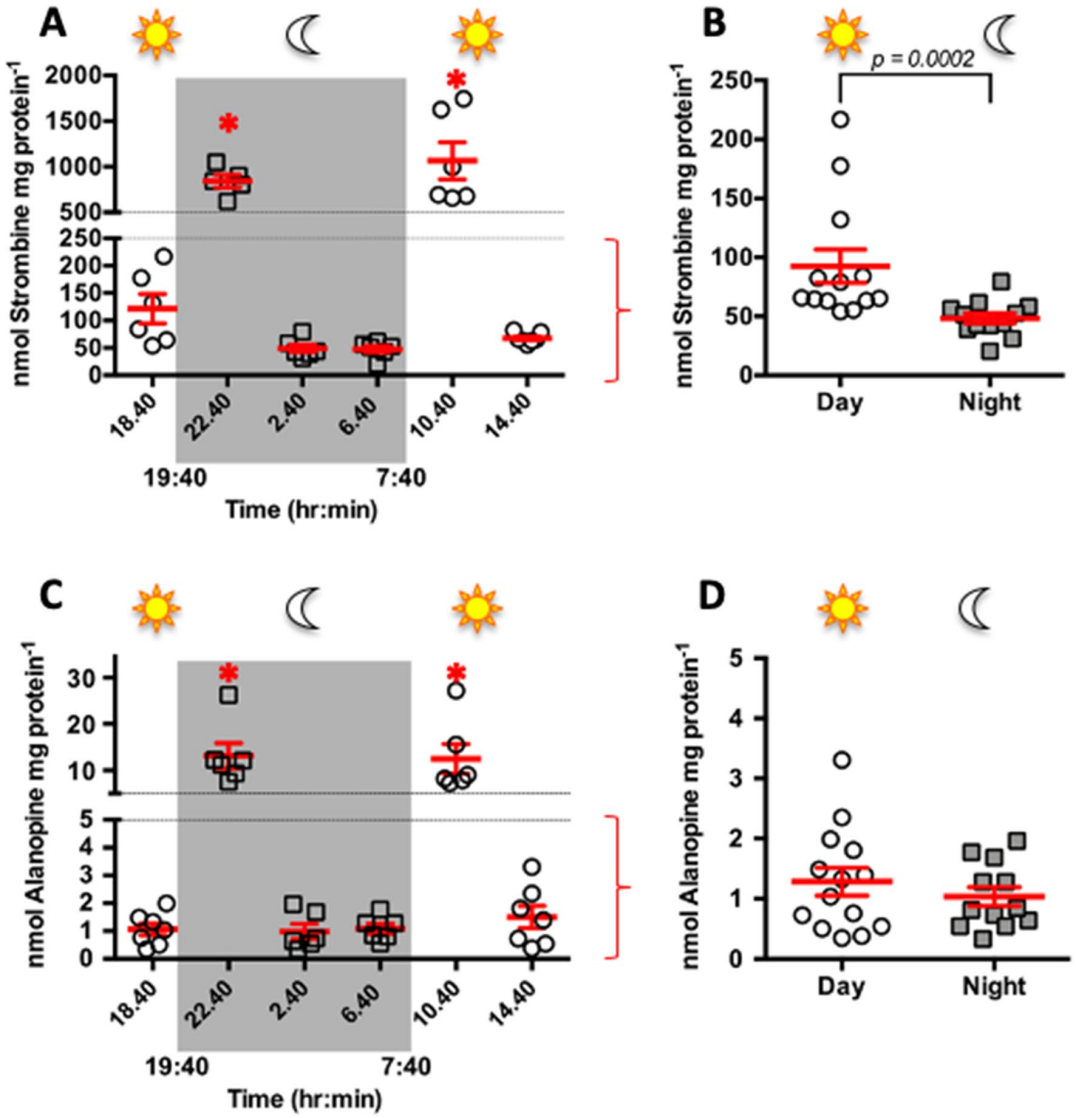
Strombine and alanopine abundances in coral tissues throughout a diel cycle. (**A**) Strombine abundance at the different time points (n = 6–7). (**B**) Pooled values from the day times 14.40 and 18.40 h were compared to pooled values from the night times 2.40 and 6.40 h (n = 12–13). The peaks at 10.40 and 6.40 h were omitted in this analysis. (**C**) Alanopine abundance at the different time points (n = 6–7). (**D**) Same as in (**B**), but for alanopine (n = 12–14). Data in (**A**) and (**C**) was squared-root transformed and analyzed by one-way ANOVA (*p* < 0.0001) followed by Tukey post-test (which corrects for multiple comparisons) (*p* < 0.05). Data in (**B**) was analyzed by Mann–Whitney test (*p* = 0.0002). Data in (**D**) was analyzed by unpaired t-test (*p* = 0.25).

Having identified strombine as the main end-product of coral fermentation, we were able to explore the use of this pathway throughout a 24 h period by quantifying strombine levels in *A. yongei* tissues using MRM LC–MS. Strombine concentrations in *A. yong*ei ranged from ~ 20 to 1744 nmol mg protein^−1^ (Fig. 4), which are amongst the highest levels ever reported in a marine invertebrate. As a reference*,* the posterior adductor muscle of *M. edulis* accumulated ~ 30 nmol strombine mg protein^-1^ during normal conditions, ~ 100 nmol strombine mg protein^−1^ after 24 h hypoxia induced by aerial exposure, and a maximum of ~ 250 nmol strombine mg protein^−1^ four hours into the subsequent recovery period following re-submersion in seawater (estimated after converting “g wet weight” from^43^ into “mg protein” using protein concentrations found in^44^).

Acroporid corals have relatively thin tissues with small diffusional distances, and thus [O_2_] measurements in their DBL accurately reflect [O_2_] in the coral cells^5^. We predicted that nightly hypoxia would be associated with increased reliance on fermentation and associated increased strombine and alanopine production. However, time point analyses revealed a more complex picture. The most apparent feature was the presence of distinct peaks in strombine and alanopine concentrations at the first sampling points following the transitions between light–dark and dark–light (Fig. 4A,C) corresponding to the onsets of hypoxia and hyperoxia (Fig. 1), respectively. Importantly, these sampling points were 3 h after the prior change in illumination conditions. Opine concentrations in these peaks were 987.9 ± 155.5 and 1065 ± 202.8 nmol mg protein^−1^ and 13.1 ± 2.7 and 12.5 ± 3.2 nmol alanopine. mg protein^−1^, at the early night and early day time points, respectively. The peaks in opine production at the beginning of the night followed the reduction in DBL [O_2_] after photosynthesis had shut down and coral cells, symbiotic algae, and coral-associated microbes continued consuming O_2_, but while there likely still was an abundance of photosynthetically fixed carbon. This resembles the Pasteur effect^45,46^, whereby increased glycolytic rates under O_2_-limiting and glucose-rich conditions helps sustain relatively high ATP production rates. Conversely, the strombine peak at the first morning time point may be due to the sudden availability of both O_2_ and photosynthates, which allows paying the O_2_ debt incurred during the night. Similar patterns and explanations apply to intertidal invertebrates while they transition between normoxia and hypoxia during aerial exposure and re-immersion^14,19,43^.

Moreover, analyses of strombine concentrations between corals sampled during day and night, but without including the peaks, revealed that the former produced nearly double the amount of strombine (92.5 ± 14.2 vs. 48.3 ± 4.4 nmol mg protein^−1^, respectively, *p* < 0.0005, unpaired t-test) (Fig. 4B). On the other hand, no such differences were detected for alanopine (1.3 ± 0.2 and 1.0 ± 0.2 nmol mg protein^-1^ during day and night) (Fig. 4D). These results indicate that, on average, corals use SDH to regenerate NAD^+^ and produce ATP at a higher rate during the day compared to the night, despite experiencing hyperoxia and hypoxia, respectively. We tentatively propose that this is related to the overabundance of photosynthates during the day, which can be used as metabolic fuel^47,48^. Indeed, although fermentation is less efficient than aerobic respiration, it can take place at much faster rates, so it may be favored under nutrient-rich conditions even in the presence of O_2_^49^. This aerobic fermentation is known as the Warburg effect and was originally described in cancer cells^49^, and has more recently been identified in a variety of other proliferative cell types^50^.

A final consideration is that aerobic respiration rates in corals exposed to light can be up to seven times higher compared to corals exposed to darkness^5,7^. This implies that although the rate of strombine production is lower at nighttime compared to daytime, its relative contribution to overall ATP production must be greater during the former. With these caveats in mind, corals’ reliance on fermentation may actually increase during nightly hypoxia.

## Conclusions

The current study determined prevalent hyperoxic conditions in the coral DBL during the day and average hypoxic conditions during the night. Studying potential changes in anaerobic metabolism required a better understanding of *A. yongei* fermentation, which led to the identification of strombine as corals’ main fermentative end-product, and of SDH as the main terminal dehydrogenase. Because these results align with the conclusions from a previous study on a species of brain coral, *M. capitata*^17^, the importance of fermentation throughout day and night likely applies more broadly to reef-building corals. The dynamic changes in strombine concentration in coral tissues throughout a diel cycle despite relatively constant levels of metabolic enzymes indicate that adjustments to ATP production are largely modulated by the immediate availability of glucose (and pyruvate) derived from photosynthesis compounded by O_2_ availability. The availability of other metabolic substrates such as glycine and alanine as well as the NADH/NAD^+^ ratio could also determine opine production rates. Additionally, the activities of the relevant enzymes could be subjected to post-translational regulation including phosphorylation, acetylation, and availability of divalent metals that may act as cofactors. These are all interesting topics for future research.

In our laboratory experiments, the dynamic changes in fermentative activity were evidenced by a higher average strombine concentration at daytime compared to nighttime (reminiscent of the Warburg effect), and by pronounced peaks in strombine concentration early in the night and early in the morning. Due to the continuous and rapid changes in light and water flow in natural coral reef environments, the changes in fermentative rates in corals in situ are poised to be even more dynamic. Moreover, coral fermentation rates are likely to be affected by feeding, a parameter that was not controlled in our experiments.

In addition to short-term and diel O_2_ variability, hypoxia and anoxia might develop at coral-algal interaction zones^51–53^, underneath toxic algal mats^54^, during coral-microbial interactions^55^, during natural upwelling events, and in increasingly frequent ‘dead zones’ of anthropogenic origin^56^. Thus, the novel findings about coral energy metabolism presented in the current study could have far-reaching implications for the energy metabolism of healthy and stressed corals.

## Methods

### Specimens and experimental aquaria

Branch tips of *Acropora yongei* (~ 4 cm) originated from individuals obtained from the Birch Aquarium at the Scripps Institution of Oceanography (SIO) (San Diego, CA, USA) and were grown for years at the SIO Tropical Experimental Aquarium facility. These coral colonies predominantly host *Cladocopium sp.* dinoflagellate algae. After sectioning from the main colonies using bone cutters, the tips were glued onto ceramic tiles and allowed to recover for 6 weeks in a 75-L tank with 18–20 μm filtered flow-through seawater piped from the SIO Research Pier and heated to 26 °C (± 1 °C). Zooplankton availability, which determines the rate of coral heterotrophic feeding, was not controlled and paralleled that in the seawater piped from the SIO pier (but diminished due to filtering). The average phosphate (PO_4_^3−^), nitrate (NO_3_^-^), and ammonium (NH_4_^+^) concentrations during the September 2014 experiments were 0 μM, 0 μM, and 0.6 ± 0.2 μM, respectively (Southern California Coastal Ocean Observing System; https://sccoos.org/harmful-algal-bloom/). The average PO_4_^3−^, NO_3_^−^, and NH_4_^+^ concentrations during the April 2016 experiments were 0 μM, 0.5 ± 0.5 μM, and 0 μM, respectively.

Coral tips were maintained under photoperiods set to a 12 h:12 h (2014) or 13 h:11 h (2016) light:dark cycle. Average photosynthetically active radiation (PAR) at the coral branch tips was ~ 200 μmol photons m^−2^ s^−1^. Seawater was circulated within the aquaria using two flow pumps (908 L h^−1^, Hydor Koralia Nano 3.5 W Aquarium Circulation Pumps) positioned on opposite sides of the tanks, with inflowing seawater distributed by eight 6.35 mm plastic tubes spaced evenly around the sides of the tanks. Homogeneous and sufficient mixing throughout the tanks was confirmed using neutrally buoyant tracer particles illuminated using a laser sheet in the dark^57^.

Fish samples were used in the study as positive controls for the LDH assay, which were obtained according to protocol no. S10320 approved by SIO-UCSD animal care committee in compliance with the IACUC guidelines for the care and use of experimental animals.

### Microsensor measurements and coral sampling

O_2_ concentrations ([O_2_]) on the coral tissue surface in the diffusive boundary layer (DBL) were measured using Clark-type microsensors (tip diameter: 100 μm; stirring sensitivity: 1.5%, 90% response time: < 8 s; Unisense A/S, Aarhus, Denmark) and recorded using the Unisense SensorTrace Logger software with a recording rate of 60 s^−1^. Microsensors were calibrated daily using fully aerated seawater and an anoxic solution (0.1 M sodium ascorbate and 0.1 M NaOH). The microsensor was carefully positioned on the coral tissue surface using a manual micromanipulator (Unisense A/S, Aarhus, Denmark), under observation using a binocular dissection microscope. Measurements were conducted on the coenosarc to minimize interference from polyp movement^5^. [O_2_] measurements in the DBLs were continuously recorded for at least 16 h to capture daytime and nighttime concentrations. An initial experiment conducted in September 2014 measured DBL [O_2_] on four randomly chosen branches on random nights between September 9 and October 1. On the fifth day, 6–7 coral branches were randomly sampled from the tank at the following times: 6.40, 10:40, 14:40, 18.40, 22:40, and 2:40 h. Sampling at 6.40 h took place 1 h before the lights turned on (at 7:40 h), and sampling at 18.40 h took place 1 h before the lights turned off (at 19.40 h). Coral branches were quickly drip-dried to remove excess seawater and mucus, snap frozen in liquid N_2_, and stored at −80 °C until processed for enzymatic assays, opine identification and quantification, and proteomics. A second experiment conducted in April 2016 measured [O_2_] on 7 coral branches held in a similar experimental tank set-up. Simultaneous measurements of salinity-corrected dissolved [O_2_] in the seawater surrounding the corals were made using a HQ40d portable meter with a LDO101 rugged optical dissolved O_2_ probe (Hach, Loveland, CO, CA).

### Coral tissue homogenization

To remove coral tissue from the skeleton, buffer (100 mM Tris, pH 7.5) was blasted at frozen *A. yongei* branches using an airbrush (Paasche, MIL#3 Millennium Airbrush). To shear the mucus, coral homogenates were vortexed and syringed using a 21 gauge needle, on ice. Homogenate aliquots were frozen in liquid N_2_ and stored at −80 °C until the various assays (i.e. enzyme activity, protein and metabolite concentrations) were performed. Protease (Protease Inhibitor Cocktail, Sigma, MO) and phosphatase (PhosSTOP, Roche) cocktails were added to aliquots used for enzyme activity assays, at the concentrations recommended by the manufacturers.

### Metabolic enzyme activity assays

Enzymatic assays for CS (E.C. 2.3.3.1), MDH (E.C. 1.1.1.37), SDH (E.C. 1.5.1.22), ADH (E.C. 1.5.1.17), and LDH (E.C. 1.1.1.27) were performed in duplicates on a microplate reader (Spectra Max M2, Molecular Devices) at 28 °C in 96-well plates (Costar 96-Well EIA/RIA plates, Corning). The final reaction volume was 160 μL. Preliminary experiments revealed that the pellet containing the algal symbionts did not have detectable activity for any of the enzymes measured in this study. Indeed, measuring enzymatic activity or symbionts’ metabolites requires harsh disruption techniques using a bead-beater^27,58^. To preserve gastrodermal cells that would also be lost during centrifugation, all subsequent assays were performed on total tissue homogenates and enzymatic activity was assumed to reflect that of the coral host. Enzymatic reactions were initiated by the addition of the assay mix to the coral tissue homogenate. All assays were optimized for coral tissues. CS activity was measured in an assay medium with final concentrations of 2.0 mM MgCl_2_, 0.1 mM DTNB, 0.1 mM acetyl CoA, 80 mM Tris buffer (pH 8.0 at 20 °C), and 0.5 mM oxaloacetate. MDH activity was measured using established methods^30^, using final concentrations of 80 mM imidazole-HCl buffer (pH 7.0 at 20 °C), 100 mM KCl, 0.3 mM oxaloacetate, and 0.15 mM NADH. LDH activity was measured in 0.15 mM NADH, 100 mM KCl, 50 mM imidazole buffer (pH 7.0 at 20 °C), and 1.0 mM pyruvate. Positive controls were run using pure L-LDH and fish white muscle homogenates, which yielded the expected activity^59^. The SDH assay was adapted from a previous study^60^, using final concentrations of 100 mM Tris-HCl (pH 7.0 at 20 °C), 100 mM glycine, 0.3 mM Na-pyruvate, and 0.3 mM NADH. The ADH assay medium contained final concentrations of 0.2 mM NADH, 3.0 mM pyruvic acid, 200 mM alanine, and 100 mM imidazole-HCl (pH 7.0 at 20 °C), following a previous study^61^. The activities of MDH, LDH, SDH, and ADH were calculated based on the decrease in absorbance at 340 nm, while the activity of CS was calculated based on the increase in absorbance at 412 nm.

To calculate enzyme activity, the slope corresponding to the change in absorbance over the most linear range of the reaction was calculated, and each set of duplicate reactions was averaged. Homogenization buffer blanks (100 mM Tris, pH 7.5) were run alongside samples and the resulting “background” activity was subtracted. Negative controls consisting of denatured coral homogenates (90 °C for 10 min) yielded no detectable enzyme activity. All enzyme activities were standardized to total soluble protein concentration, measured using the Bradford assay (Bio-Rad Protein Assay kit). A few enzyme activity values were marginally negative after blanks were subtracted, and were recorded as zero.

### Preparation of coral extracts for liquid chromatography

To extract coral metabolites, ice-cold methanol was added to aliquots of frozen tissue homogenates collected during the 2014 diel study in a ratio of 4:1 (v/v). After sonicating the coral extracts on ice for 2 min, they were freeze-thawed in liquid N_2_ three times, and centrifuged (13,000 *g*, 15 min, 4 °C). The pellet, which contains insoluble materials and proteins, was discarded, and the supernatant was transferred to another container and evaporated to dryness using a Rotary Evaporator (Buchi R-210 Rotavapor Evaporator). To further remove insoluble debris, the dried extracts were reconstituted in MeOH:H_2_O (1:1, v/v), vortexed, and centrifuged (13,000 g, 15 min, 4 °C). The supernatants were stored at −80 °C until LC–MS analyses were performed. Additional *A. yongei* branches processed in an identical manner were used in assays designed to confirm the identity of the corals’ strombine-like compound.

### LC–MS/MS

Detailed procedures can be found in Dr. Linsmayer’s PhD thesis^33^. Briefly, alanopine, octopine, and R-strombine standards were kindly provided by Dr. W. Ross Ellington (Florida State University). The S-strombine standard was purchased from AEchem Scientific Corporation (Illinois, USA). LC–MS analyses determined that the retention times and mass spectra of R- and S-strombine isomers were identical. Pure S-strombine was used as the standard in most subsequent LC–MS analyses, simply because it was more readily available. Nopaline was obtained from Toronto Research Chemicals (Ontario, Canada). This opine is produced by *Agrobacterium* bacteria^62^, and was included in the analyses to examine potential opine production by bacteria associated with corals.

Coral extracts sampled throughout the diel cycle were analyzed using Multiple Reaction Monitoring (MRM) on a highly sensitive triple quadrupole (QqQ) mass spectrometer (6490 QqQ LC–MS system equipped with iFunnel technology; Agilent Technologies) at The Scripps Research Institute (TSRI; La Jolla, CA, USA). Coral extracts were separated in a 2.0 × 150 mm Luna amino column (Phenomenex, Torrance, CA, USA) using a mobile phase consisting of (A) 100 mM ammonium formate and (B) 100% acetonitrile (ACN). After applying a linear gradient elution from 100% B (0–1 min) to 100% A (12–16 min), a 10 min post-run ensued to ensure column re-equilibration and maintain reproducibility. Opine standards (100 fg, 1 pg, 10 pg, 100 pg, and 1000 pg) were created immediately prior to the analyses and run alongside the coral extracts. To guard against carryover, 1:1 MeOH:H_2_O blanks were run in between samples. The injection volume for all samples was 5 μL, and the flow rate was 200 μL min^−1^. Opines in the coral extracts were quantified in positive mode using MRM of the transitions of *m/z* 162 → 116 (alanopine), *m/z* 247.1 → 141.8 (octopine), *m/z* 148.6 → 102 (strombine), and *m/z* 305.1 → 200 (nopaline). A second set of transitions was used as a qualifier for confirmation, with *m/z* 162 → 69.9 (alanopine), *m/z* 247.1 → 69.9 (octopine), *m/z* 148.6 → 56 (strombine), and *m/z* 305.1 → 70 (nopaline).

To confirm of the identity of the coral strombine-like compound, dried coral extract and R-strombine standard were reconstituted in methanol, separated using a 1.0 × 150 mm Luna amino column (Phenomenex) and analyzed on an Agilent 6538 ultra high definition (UHD) Quadrupole time-of-flight (Q-TOF) mass spectrometer coupled to a 1100/1200 LC stack (Agilent) at TSRI. The mobile phase consisted of (A) 20 mM ammonium acetate + 40 mM ammonium hydroxide (95% in water), and (B) 95% ACN (in H_2_O). After applying an elution gradient of 100% B (0–5 min) to 0% B (45–55 min) to 100% B (60 min), a 9 min postrun ensued to re-equilibrate the column. The injection volume was 8 μL, and the flow rate was 50 μL min^−1^. These analyses were run in negative mode, using a MS scan range between 70–550 *m/z* and a MS/MS scan range between 25 and 200 *m/z*; collision energy was 20 V.

To even further confirm the presence of strombine in coral extracts, we used an additional LC–MS platform at UCSD after filtering samples and standards using Celite (a diatomaceous earth commonly used to filter out metal salts). Dried extracts from corals sampled during the day and 100 ng S-strombine standard were reconstituted in 100% methanol, desalted using cotton and Celite columns (Sigma-Aldrich), evaporated, and stored at − 80 °C. After reconstitution in 0.1% formic acid, coral extracts and standards were loaded onto an Agilent 1260 LC system coupled with a Thermo LCQdeca mass spectrometer. Samples were separated on an Imtakt Scherzo SM-C-18 column (2.0 mm ID × 150 mm length, 3.0 µm particle size). The mobile phase consisted of (A) 0.1% formic acid, and (B) ACN. Elution was performed at a flow rate of 0.30 mL min^-1^ over the following 18 min gradient: 0 min: 0% B; 6 min: 20% B; 8 min: 95% B; 10 min: 95% B; 11 min: 0% B, and 18 min: 0% B. LC–MS/MS analysis was run in negative ion mode using electrospray ionization (ESI) as the ion source (voltage: − 4.5 kV, sheath gas flow rate: 80 units, auxiliary gas flow rate: 20 units, capillary temperature: 250 °C).

### Proteomics

Aliquots of frozen coral tissue homogenates from the 2014 diel study were transported on dry ice to the California Polytechnic State University (Cal Poly, San Luis Obispo, CA) for 2D gel and MS enabled proteomics using a previously published workflow^63^ (also see the Supplementary Information of this manuscript). Briefly, after tissue homogenates were twice-precipitated to concentrate proteins from coral host and symbiotic algae, two sets of 2D gels were run on the same coral homogenates, under different isoelectric focusing conditions and gel stains. The first set of gels were run with a broad pH range (pH 3–10) and Coomassie Blue dye, in order to capture as much of the coral proteome as possible. Because most proteins clustered in the middle-pH range of the Coomassie-stained gels and were difficult to resolve, a second set of gels were run with a narrower isoelectric focusing range (pH 4–7) and stained with the highly sensitive fluorescent dye, SYPRO Ruby. Digitized images of gels were analyzed with Delta2D image software (version 3.6; Decodon, Greifswald, Germany). Protein spot volumes were normalized against total spot volume of all protein spots of the first gel image in the set (normalized spot volumes, NSVs). Following statistical analyses in Delta2D, all visually detectable proteins were excised from representative gels of the Coomassie and SYPRO gel sets, destained, digested, and extracted. The digested proteins were spotted on an Anchorchip target plate (Bruker Daltonics Inc., Billerica, MA, USA), washed, and recrystallized before processing in a MALDI TOF-TOF mass spectrometer (Ultraflex II; Bruker Daltonics Inc.) to obtain peptide mass fingerprints. Post-processing and identification of proteins were done as previously described^63^. Protein sequences were searched against all available cnidarian and Symbiodiniaceae databases at the time of the study.

### Statistical analyses

Data is presented as mean ± SEM. DBL [O_2_], enzyme activities and opine abundances were analyzed using one-way analysis of variance (ANOVA), t-test, or Mann–Whitney test as described in the figure legends, in Prism 7 (GraphPad Software, La Jolla, CA). Proteomic data were analyzed in Delta2D as described in detail in the Supplementary Information.

## Supplementary information

Supplementary Information.
