# Supplementary material for: Dynamic regulation of coral energy metabolism throughout the diel cycle
Source: Sci Rep. 2020 Nov 16;10:19881. doi: 10.1038/s41598-020-76828-2 (PMC7669893; doi:10.1038/s41598-020-76828-2)
Supplement: Supplementary file 1 — Supplementary Information. [file 41598_2020_76828_MOESM1_ESM.docx]

Supplementary information

**Dynamic regulation of coral energy metabolism throughout the diel cycle**

Linsmayer, Lauren Buckley; Deheyn, Dimitri Dominique; Tomanek, Lars; and Tresguerres, Martin

*Sample preparation for untargeted proteomics:*

Aliquots of frozen tissue homogenates from the diel study were transported to the California Polytechnic State University (Cal Poly) on dry ice for 2D gel and MS coupled proteomics using a previously described general workflow^1^.

To solubilize and precipitate proteins out of the samples prior to separating them on 2D gels, rehydration buffer [7M urea, 2M thiourea, 2% CHAPS (cholamidopropyl-dimethylammonio-propanesulfonic acid), 2% NP-40 (nonyl phenoxylpolyethoxylethanol-40), 0.002% Bromophenol Blue, 0.5% IPG buffer, and 100mM dithioerythritol] was added to each thawed tissue homogenate (1.2:1 v:v), vortexed, and allowed to sit at room temperature. Samples were split into aliquots and 10% trichloroacetic acid in acetone was added to each (4:1 v:v), and stored at -20°C overnight to precipitate proteins. The next day, samples were centrifuged at 4°C at 18,000 *g* for 15 min. The supernatant was decanted, and the remaining protein pellets were washed with 100% ice-cold acetone, vortexed, and spun down as before. Rehydration buffer (0.40 mL) was added to an aliquot of washed, precipitated proteins and passed through the other aliquots to concentrate proteins from the same sample.

Protein assays were run on the precipitated, concentrated samples using the 2D Quant Kit (GE Healthcare) to determine the amount of active rehydration buffer to add in order to load 400 μg of protein per gel for Coomassie staining. After running Coomassie gels, it was determined that an additional protein precipitation step was required to ensure proteins separated in the 1^st^ dimension. Thus, protein samples were twice-precipitated, and 350 μg protein were loaded onto IPG strips for later SYPRO staining.

*Two-dimensional gel electrophoresis:*

Two sets of 2D gels were run on the same coral homogenates, under different isoelectric focusing conditions and gel stains to increase capture of the coral proteome. The first set of gels were run with a broad pH range (pH 3-10) and stained with Coomassie Blue dye G-250 (Thermo Fisher Scientific, Waltham, MA, USA). A second set of gels were run with a narrower isoelectric focusing range (pH 4-7) and stained with the highly sensitive fluorescent dye, SYPRO Ruby (Invitrogen, Carlsbad, CA, USA). To separate proteins by isoelectric point, isoelectric focusing for both gel sets started with a 5h passive rehydration step, followed by 12 h active rehydration (50 V) using an isoelectric focusing cell (BioRad, Hercules, CA, USA). The following running conditions were used for the remainder of the isoelectric focusing run: 500 V for 1 hr, 1000 V for 1 hr and 8000 V for 2.5 hr (all changes occurred in rapid mode). Following isoelectric focusing, gel strips were frozen at –80°C.

To separate proteins by molecular mass, frozen IPG strips were thawed and incubated in equilibration buffer [375 mM Tris-base, 6 mM urea, 30% glycerol, 2% sodium dodecyl sulfate (SDS), 0.0002% Bromophenol Blue] for 15 min, first with 65 mM dithiothreitol and then, after decanting the solution, with 135 mM iodoacetamide in equilibration buffer. IPG strips were placed on top of an 11.8% polyacrylamide gel with a 0.8% agarose solution containing Laemmli SDS electrophoresis buffer (25 mM Tris-base, 192 mM glycine, 0.1% SDS). Gels were run at 200 V for 55 min with a recirculating water bath set at 10°C using Criterion Dodeca cells (BioRad).

The first set of gels (pH 3-10) were stained with colloidal Coomassie Blue dye overnight on a shaker and destained by washing repeatedly with Milli-Q water for at least 48 hr. The second set of gels (pH 4-7) was fixed stained in 7% acetic acid/50% methanol and stained with SYPRO Ruby stain in covered containers overnight. SYPRO gel destaining was done in 10% methanol and 7% acetic acid.

The Coomassie-stained gels were scanned with an Epson 1280 transparency scanner (Epson, Long Beach, CA, USA), and the SYPRO Ruby-stained gels were scanned in the dark with a Typhoon Trio+ Imager (GE Healthcare, Piscataway, NJ, USA) at 700 pmt voltage and a laser path of 50 μm.

*Gel image analysis:*

Digitized images of gels were analyzed with Delta2D image software (version 3.6; Decodon, Greifswald, Germany). Based on proteins in some samples not fully separating during isoelectric focusing, six gels from the Coomassie set and one from the SYPRO set were excluded from warping and analysis. The remaining gels were compared with each other using the group warping strategy by creating match vectors between the gels within a treatment group and then the first gels between each treatment. All images from each gel group were fused into a composite image (i.e. “proteome map”), which represents mean volumes for each protein spot. The proteome map was used to detect spot boundaries, which were subsequently transferred back to all gel images using previously generated match vectors. After background subtraction, protein spot volumes were normalized against total spot volume of all protein spots of the first gel image in the set (normalized spot volumes, NSVs).

*Mass spectrometry for proteomics:*

Following statistical analyses in D2D, all visually detectable proteins were excised from representative gels of the Coomassie and SYPRO gel sets using a tissue puncher (Beecher Instruments, Prairie, WI, USA). The excised gel plugs were destained by two washes with 25 mM ammonium bicarbonate in 50% acetonitrile before dehydration with 100% acetonitrile and subsequent digestion with 11 ng μL^-1^ trypsin (Promega, Madison, WI, USA) overnight at 37°C. Digested proteins were extracted in an elution buffer [0.1% trifluoroacetic acid (TFA): acetonitrile 2:1] and concentrated in a SpeedVac (Thermo Fisher Scientific, Waltham, MA, USA). The digested proteins in elution buffer were mixed with 5 μL of matrix solution (0.2 mg mL^–1^ α-Cyano-4-hydroxycinnamic acid in acetonitrile) and spotted on an Anchorchip® target plate (Bruker Daltonics Inc., Billerica, MA, USA). Proteins spotted to the target were washed with 0.1% TFA in 10 mM ammonium phosphate and re-crystallized using an acetone:ethanol:0.1% TFA (6:3:1 v:v:v) mixture. Peptide mass fingerprints (PMFs) were obtained on a matrix assisted laser desorption ionization tandem time-of-flight (MALDI TOF-TOF) mass spectrometer (Ultraflex II; Bruker Daltonics Inc.). We selected a minimum of six peptides for tandem mass spectrometry in order to obtain information about the b- and y-ions of the peptide sequence for use in subsequent protein identification.

Mass spectra were analyzed with flexAnalysis (version 3.0; Bruker Daltonics Inc.) by applying the following conditions: TopHat algorithm for baseline subtraction, Savitzky-Golay analysis for smoothing (0.2 *m/z*; number of cycles=1) and SNAP algorithm for peak detection (signal-to-noise ratio: 6 for MS and 1.5 for MS/MS). The charge state of the peptides was assumed to be +1. Porcine trypsin was used for internal mass calibration.

Proteins were identified using Mascot (version 2.2; Matrix Science Inc., Boston, MA, USA) by combining PMFs and tandem mass spectra in a search against all publicly available cnidarian and *Symbiodinium* sequence databases at the time of the study. The cnidarian and *Symbiodinium* databases used contained expressed sequence tags (ESTs) from *Acropora millepora* (NCBI); *Acropora hyacinthus* (Palumbi Lab, Stanford University, CA); *Aiptasia pallida*, *Anemonia viridis*, *Acropora palmata*, *Monastrea faveolata*, *Porites asteroides* (last 5 from [www.compagen.org](http://www.compagen.org)); *Symbiodinium* clades A, B, C, and D (Medina Lab, The Pennsylvania State University, PA); and *Vibrio coralliilyticus* (NCBI). Predicted protein libraries from *Acropora digitifera* (courtesy Dr. David Stead, Aberdeen Proteomics, University of Aberdeen, Scotland), *Symbiodinium* clade B1 (marinegenomics.oist.jp), and *Pocillopora damicornis* (<http://cnidarians.bu.edu/PocilloporaBase/>) were also searched. Once searching specific databases, a general search of all Metazoan EST libraries in NCBI was done. We chose oxidation of methionine and carbamidomethylation of cysteine as variable modifications, and allowed for one missed cleavage during trypsin digestion. For MS/MS, we set the precursor-ion mass tolerance to 0.6 Da. Individual molecular weight search (MOWSE) ion scores that indicated significant identity (*p*<0.05) depended on the database it matched. Our search results that were obtained with the EST and protein databases were tested against a decoy database (using Mascot) and resulted in no detection of false positives. However, we only accepted positive identifications that included two matched peptides regardless of the MOWSE score.

*Proteomics statistical analysis*

Gel images were digitized and warped to create an average gel image each for the Coomassie and SYPRO gel sets, which were used for statistics in Delta2D. Normalized spot volumes (NSVs) were analyzed using a one-way analysis of variance (one-way ANOVA) with sampling time as the main effect. The one-way ANOVA was based on a null distribution that was generated using 1000 permutations of the data, to account for any non-normal distributions and unequal variances. A *p*-value of 0.02 was used to limit the false positive discovery rate. Additional *post-hoc* analyses were conducted using Tukey’s honestly significant differences test (p<0.05) in GraphPad Prism (version 7; GraphPad Software Inc., La Jolla, CA, USA).

**References**

1. Tomanek, L. & Zuzow, M. J. The proteomic response of the mussel congeners *Mytilus galloprovincialis* and *M. trossulus* to acute heat stress: implications for thermal tolerance limits and metabolic costs of thermal stress. *The Journal of Experimental Biology* **213**, 3559–3574 (2010).

**Table S1: Diel coral and *Symbiodinium* protein expression.** *Acropora yongei* and *Symbiodinium* protein abundance over the diel cycle. Proteins were identified in Coomassie and SYPRO stained gels (see Methods for details). Proteins involved in energy metabolic pathways are **in bold**. Proteins that exhibited significant changes in diel expression (1000 permutations ANOVA, p<0.02) are indicated. Proteins that are listed more than once were identified in different gel spots, and therefore are protein isoforms. Data is shown as mean normalized spot volumes (standard errors are shown in parentheses next to means).

| **Coral Proteins That Do Not Show Significant Changes in Abundance** | | | | | | |
| --- | --- | --- | --- | --- | --- | --- |
|  | | | | | | |
|  | **18:40** | **22:40** | **02:40** | **6:40** | **10:40** | **14:40** |
| Actin | 2.52 (0.19) | 2.84 (0.39) | 2.72 (16) | 2.00 (0.32) | 2.66 (0.29) | 2.42 (0.14) |
| Actin | 1.51 (0.19) | 1.97 (0.31) | 1.40 (0.09) | 1.22 (0.11) | 1.52 (0.12) | 1.64 (0.08) |
| Actin | 0.74 (0.11) | 1.01 (0.20) | 0.89 (0.14) | 0.81 (0.11) | 0.72 (0.07) | 0.85 (0.11) |
| Alpha-tubulin | 0.28 (0.04) | 0.53 (0.14) | 0.35 (0.11) | 0.50 (0.07) | 0.42 (0.10) | 0.39 (0.11) |
| Alpha-tubulin | 0.26 (0.04) | 0.41 (0.13) | 0.35 (0.10) | 0.48 (0.12) | 0.33 (0.09) | 0.33 (0.10) |
| Beta gamma crystallin isoform 9 | 0.59 (0.12) | 0.66 (0.02) | 0.64 (0.06) | 0.58 (0.05) | 0.70 (0.04) | 0.74 (0.08) |
| Beta-actin | 1.40 (0.15) | 1.90 (0.28) | 1.57 (0.16) | 1.73 (0.13) | 1.64 (0.17) | 1.65 (0.13) |
| Beta-tubulin | 0.91 (0.18) | 1.32 (0.41) | 0.97 (0.26) | 1.05 (0.23) | 0.77 (0.20) | 0.99 (0.44) |
| Beta-tubulin | 1.07 (0.12) | 2.08 (0.44) | 1.60 (0.24) | 2.01 (0.21) | 1.62 (0.28) | 1.40 (0.28) |
| Beta-tubulin | 2.38 (0.45) | 2.34 (0.37) | 1.89 (0.50) | 2.61 (0.59) | 2.55 (0.37) | 1.38 (0.12) |
| C-type lectin protein | 0.27 (0.04) | 0.33 (0.07) | 0.19 (0.09) | 0.38 (0.11) | 0.20 (0.05) | 0.28 (0.04) |
| Choloylglycine hydrolase family protein | 0.71 (0.05) | 0.52 (0.11) | 0.52 (0.11) | 0.57 (0.07) | 0.53 (0.09) | 0.71 (0.07) |
| Choloylglycine hydrolase family protein | 0.33 (0.02) | 0.21 (0.05) | 0.39 (0.10) | 0.22 (0.08) | 0.28 (0.03) | 0.38 (0.06) |
| Choloylglycine hydrolase family protein | 0.82 (0.06) | 0.80 (0.06) | 0.72 (0.05) | 0.75 (0.06) | 0.83 (0.08) | 0.83 (0.08) |
| Choloylglycine hydrolase family protein | 0.80 (0.07) | 0.67 (0.06) | 0.65 (0.06) | 0.56 (0.05) | 0.68 (0.07) | 0.65 (0.12) |
| Cytoplasmic actin | 0.60 (0.12) | 0.83 (0.16) | 0.60 (0.05) | 0.48 (0.08) | 0.73 (0.08) | 0.81 (0.08) |
| Endonuclease III | 0.14 (0.05) | 0.07 (0.01) | 0.07 (0.01) | 0.06 (0.01) | 0.07 (0.01) | 0.10 (0.02) |
| Epididymal secretory protein E1 precursor | 0.71 (0.04) | 0.54 (0.03) | 0.56 (0.06) | 0.58 (0.03) | 0.60 (0.03) | 0.57 (0.04) |
| F-actin capping protein subunit alpha | 0.19 (0.01) | 0.16 (0.03) | 0.20 (0.03) | 0.22 (0.03) | 0.29 (0.08) | 0.22 (0.02) |
| **Fructose-bisphosphate aldolase** | 0.30 (0.03) | 0.18 (0.01) | 0.30 (0.03) | 0.26 (0.10) | 0.32 (0.06) | 0.22 (0.05) |
| **Fructose-bisphosphate aldolase** | 0.32 (0.03) | 0.29 (0.03) | 0.26 (0.03) | 0.34 (0.04) | 0.34 (0.02) | 0.35 (0.02) |
| **Fructose-bisphosphate aldolase** | 0.26 (0.02) | 0.30 (0.04) | 0.27 (0.02) | 0.26 (0.03) | 0.27 (0.03) | 0.25 (0.02) |
| Gelsolin | 0.92 (0.10) | 0.58 (0.07) | 0.89 (0.09) | 0.81 (0.10) | 0.74 (0.07) | 0.77 (0.07) |
| Gelsolin | 0.51 (0.10) | 0.40 (0.04) | 0.30 (0.04) | 0.38 (0.02) | 0.39 (0.05) | 0.42 (0.07) |
| Germ cell-less | 0.15 (0.05) | 0.11 (0.02) | 0.16 (0.05) | 0.14 (0.03) | 0.12 (0.01) | 0.13 (0.02) |
| **Glutamine synthetase** | 0.19 (0.02) | 0.20 (0.02) | 0.15 (0.01) | 0.17 (0.03) | 0.20 (0.03) | 0.21 (0.04) |
| **Glyceraldehyde-3-phosphate dehydrogenase** | 0.63 (0.05) | 0.54 (0.03) | 0.64 (0.03) | 0.55 (0.06) | 0.53 (0.02) | 0.55 (0.03) |
| **Glyceraldehyde-3-phosphate dehydrogenase** | 0.18 (0.03) | 0.20 (0.02) | 0.17 (0.03) | 0.14 (0.02) | 0.22 (0.03) | 0.20 (0.02) |
| **Glyceraldehyde-3-phosphate dehydrogenase** | 0.21 (0.04) | 0.21 (0.03) | 0.22 (0.03) | 0.18 (0.01) | 0.23 (0.03) | 0.24 (0.03) |
| Green fluorescent protein FP497 | 0.69 (0.12) | 0.80 (0.07) | 0.53 (0.07) | 0.57 (0.05) | 0.69 (0.13) | 0.81 (0.15) |
| Green fluorescent protein FP497 | 1.28 (0.11) | 1.36 (0.11) | 1.25 (0.11) | 0.97 (0.10) | 1.40 (0.16) | 1.58 (0.12) |
| Green fluorescent protein FP497 | 0.84 (0.09) | 0.83 (0.08) | 0.55 (0.02) | 0.72 (0.13) | 0.84 (0.10) | 0.91 (0.07) |
| Guanine nucleotide binding protein beta 2 | 0.15 (0.02) | 0.15 (0.03) | 0.16 (0.02) | 0.19 (0.03) | 0.19 (0.01) | 0.17 (0.01) |
| Guanine nucleotide-binding protein subunit beta | 0.39 (0.01) | 0.43 (0.03) | 0.36 (0.04) | 0.44 (0.03) | 0.44 (0.03) | 0.38 (0.07) |
| Haem peroxidase domain containing protein | 0.12 (0.03) | 0.15 (0.01) | 0.15 (0.03) | 0.16 (0.03) | 0.17 (0.03) | 0.21 (0.03) |
| Haem peroxidase domain containing protein | 0.17 (0.05) | 0.20 (0.03) | 0.18 (0.03) | 0.21 (0.05) | 0.16 (0.02) | 0.18 (0.02) |
| Heat shock cognate protein 70 | 0.41 (0.11) | 0.31 (0.06) | 0.41 (0.11) | 0.32 (0.14) | 0.30 (0.07) | 0.21 (0.06) |
| Heat shock cognate protein 70 | 0.14 (0.04) | 0.15 (0.03) | 0.17 (0.03) | 0.15 (0.05) | 0.13 (0.04) | 0.10 (0.02) |
| Heat shock protein 78 | 0.18 (0.04) | 0.15 (0.01) | 0.25 (0.07) | 0.17 (0.08) | 0.17 (0.04) | 0.11 (0.04) |
| **Isocitrate dehydrogenase [NADP], mitochondria** | 0.21 (0.08) | 0.16 (0.05) | 0.11 (0.07) | 0.15 (0.07) | 0.18 (0.04) | 0.09 (0.03) |
| Nematoblast-specific protein nb012b | 0.53 (0.08) | 0.49 (0.02) | 0.45 (0.07) | 0.59 (0.06) | 0.66 (0.05) | 0.64 (0.04) |
| Niemann-Pick C 2 Like | 0.39 (0.04) | 0.38 (0.01) | 0.31 (0.03) | 0.38 (0.05) | 0.41 (0.04) | 0.38 (0.05) |
| Pseudouridylate synthase | 0.18 (0.02) | 0.24 (0.02) | 0.22 (0.03) | 0.21 (0.06) | 0.20 (0.01) | 0.24 (0.02) |
| RecName: Full=ZP domain containing protein; Flags: Precursor | 1.11 (0.12) | 1.03 (0.19) | 1.03 (0.07) | 0.60 (0.07) | 1.16 (0.17) | 1.02 (0.09) |
| Serum albumin precursor | 0.61 (0.08) | 0.54 (0.04) | 0.39 (0.07) | 0.69 (0.11) | 0.62 (0.06) | 0.67 (0.04) |
| Severin | 0.28 (0.03) | 0.30 (0.05) | 0.27 (0.02) | 0.34 (0.07) | 0.33 (0.03) | 0.30 (0.02) |
| Sushi, von Willebrand factor type A, EGF and pentraxin domaincontaining protein 1 | 0.17 (0.02) | 0.18 (0.02) | 0.15 (0.01) | 0.16 (0.01) | 0.16 (0.02) | 0.14 (0.02) |
| TPA: fluorescent protein 2 | 3.23 (0.26) | 3.76 (0.24) | 3.19 (0.19) | 2.96 (0.20) | 3.63 (0.41) | 3.98 (0.36) |
| TPA: fluorescent protein 2 | 0.76 (0.14) | 0.83 (0.06) | 0.66 (0.13) | 0.52 (0.01) | 0.75 (0.13) | 0.81 (0.14) |
| TPA: fluorescent protein 2 | 0.14 (0.04) | 0.15 (0.02) | 0.13 (0.03) | 0.10 (0.01) | 0.19 (0.04) | 0.12 (0.03) |
| TPA: fluorescent protein 2 | 0.27 (0.05) | 0.32 (0.03) | 0.29 (0.03) | 0.21 (0.02) | 0.28 (0.05) | 0.28 (0.05) |
| TPA: fluorescent protein 2 | 1.40 (0.08) | 1.43 (0.13) | 1.45 (0.18) | 1.29 (0.21) | 1.56 (0.10) | 1.67 (0.17) |
| Voltage-dependent anion selective channel protein 2 | 0.33 (0.03) | 0.30 (0.04) | 0.32 (0.04) | 0.32 (0.09) | 0.31 (0.03) | 0.34 (0.02) |
| Voltage-dependent anion selective channel protein 2 | 0.26 (0.06) | 0.18 (0.03) | 0.18 (0.04) | 0.18 (0.01) | 0.21 (0.02) | 0.19 (0.02) |
|  |  |  |  |  |  |  |
| **Coral Proteins That Show Significant Changes in Abundance** | | | | | | |
|  | **18:40** | **22:40** | **02:40** | **6:40** | **10:40** | **14:40** |
| Na^+^/H^+^ exchange regulatory cofactor NHE-RF2 | 0.22 (0.05) | 0.12 (0.02) | 0.29 (0.10) | 0.30 (0.10) | 0.12 (0.02) | 0.06 (0.02) |
| 56kDa selenium binding protein | 0.16 (0.01) | 0.26 (0.03) | 0.23 (0.03) | 0.30 (0.02) | 0.22 (0.02) | 0.24 (0.02) |
| Actin | 0.76 (0.09) | 1.13 (0.16) | 0.64 (0.05) | 0.90 (0.04) | 0.83 (0.05) | 0.70 (0.03) |
| Actin | 1.88 (0.23) | 2.85 (0.32) | 1.97 (0.05) | 2.36 (0.15) | 2.22 (0.15) | 2.11 (0.14) |
| Actin/choloylglycine hydrolase family protein | 0.17 (0.02) | 0.14 (0.02) | 0.16 (0.03) | 0.07 (0.02) | 0.13 (0.02) | 0.07 (0.02) |
| **ATP synthase subunit beta, mitochondrial** | 0.16 (0.02) | 0.23 (0.01) | 0.19 (0.02) | 0.25 (0.03) | 0.20 (0.01) | 0.22 (0.02) |
| F-actin capping protein subunit alpha | 0.10 (0.02) | 0.01 (0.00) | 0.09 (0.01) | 0.10 (0.02) | 0.14 (0.04) | 0.11 (0.02) |
| Guanine nucleotide binding protein beta 2 | 0.11 (0.03) | 0.14 (0.02) | 0.14 (0.02) | 0.22 (0.06) | 0.09 (0.02) | 0.19 (0.01) |
|  |  |  |  |  |  |  |
| **Symbiont Proteins That Do Not Show Significant Changes in Abundance** | | | | | | |
|  | **18:40** | **22:40** | **02:40** | **6:40** | **10:40** | **14:40** |
| Alpha-tubulin | 0.83 (0.14) | 0.77 (0.14) | 0.76 (0.29) | 1.08 (0.31) | 0.76 (0.18) | 0.51 (0.07) |
| **ATP synthase beta subunit** | 0.16 (0.02) | 0.25 (0.04) | 0.16 (0.01) | 0.22 (0.03) | 0.17 (0.02) | 0.18 (0.01) |
| Chloroplast ferredoxin-NADP^+^ reductase | 0.14 (0.02) | 0.15 (0.00) | 0.11 (0.01) | 0.15 (0.01) | 0.14 (0.02) | 0.16 (0.02) |
| Chloroplast ferredoxin-NADP^+^ reductase | 0.41 (0.08) | 0.56 (0.11) | 0.46 (0.12) | 0.49 (0.06) | 0.58 (0.07) | 0.55 (0.09) |
| Chloroplast light harvesting complex protein | 0.94 (0.29) | 0.75 (0.36) | 0.61 (0.10) | 0.47 (0.11) | 0.67 (0.04) | 0.67 (0.07) |
| Chloroplast oxygen-evolving enhancer | 1.14 (0.03) | 1.16 (0.03) | 1.12 (0.12) | 1.13 (0.11) | 1.19 (0.07) | 1.24 (0.01) |
| Chloroplast oxygen-evolving enhancer | 0.13 (0.02) | 0.11 (0.01) | 0.18 (0.02) | 0.19 (0.02) | 0.15 (0.01) | 0.15 (0.02) |
| Chloroplast oxygen-evolving enhancer | 0.28 (0.04) | 0.25 (0.03) | 0.27 (0.05) | 0.33 (0.06) | 0.30 (0.04) | 0.33 (0.03) |
| Chloroplast oxygen-evolving enhancer | 1.16 (0.10) | 1.12 (0.07) | 0.89 (0.15) | 0.96 (0.13) | 1.13 (0.09) | 1.18 (0.08) |
| Chloroplast ribulose-1,5- bisphosphate carboxylase/oxygenase large subunit | 1.91 (0.12) | 1.56 (0.11) | 1.72 (0.23) | 1.41 (0.13) | 1.78 (0.11) | 1.93 (0.10) |
| Chloroplast ribulose-1,5- bisphosphate carboxylase/oxygenase large subunit | 1.92 (0.10) | 1.75 (0.05) | 1.92 (0.13) | 1.96 (0.14) | 1.99 (0.09) | 1.99 (0.06) |
| Chloroplast soluble peridinin chlorophyll a-binding protein precursor | 0.13 (0.03) | 0.06 (0.01) | 0.08 (0.02) | 0.08 (0.02) | 0.08 (0.02) | 0.07 (0.02) |
| Chloroplast soluble peridinin chlorophyll a-binding protein precursor | 0.19 (0.04) | 0.18 (0.03) | 0.22 (0.02) | 0.16 (0.02) | 0.18 (0.02) | 0.18 (0.02) |
| Chloroplast soluble peridinin chlorophyll a-binding protein precursor | 0.39 (0.04) | 0.32 (0.04) | 0.32 (0.06) | 0.28 (0.06) | 0.36 (0.04) | 0.36 (0.03) |
| Chloroplast soluble peridinin chlorophyll a-binding protein precursor | 0.43 (0.06) | 0.36 (0.04) | 0.36 (0.06) | 0.40 (0.13) | 0.40 (0.06) | 0.46 (0.04) |
| Class IVb beta tubulin | 0.70 (0.10) | 1.02 (0.26) | 0.90 (0.22) | 0.92 (0.18) | 0.74 (0.17) | 0.82 (0.27) |
| Class IVb beta tubulin | 1.85 (0.4) | 1.55 (0.39) | 1.63 (0.27) | 2.41 (0.73) | 2.01 (0.42) | 0.95 (0.13) |
| FMN-linked oxidoreductase | 0.17 (0.01) | 0.17 (0.03) | 0.12 (0.01) | 0.19 (0.02) | 0.16 (0.01) | 0.21 (0.02) |
| **Fumarate reductase** | 0.26 (0.04) | 0.33 (0.06) | 0.30 (0.12) | 0.20 (0.03) | 0.21 (0.02) | 0.23 (0.02) |
| **Fumarate reductase** | 0.29 (0.02) | 0.33 (0.07) | 0.23 (0.02) | 0.27 (0.02) | 0.29 (0.01) | 0.33 (0.02) |
| Gelsolin | 0.19 (0.09) | 0.10 (0.01) | 0.11 (0.02) | 0.11 (0.02) | 0.08 (0.01) | 0.09 (0.01) |
| **Glyceraldehyde-3-phosphate dehydrogenase** | 0.44 (0.08) | 0.42 (0.07) | 0.45 (0.03) | 0.41 (0.10) | 0.56 (0.05) | 0.62 (0.06) |
| Lamin | 0.23 (0.05) | 0.22 (0.03) | 0.24 (0.03) | 0.28 (0.05) | 0.28 (0.03) | 0.33 (0.05) |
| **Malate dehydrogenase** | 0.17 (0.02) | 0.16 (0.01) | 0.21 (0.05) | 0.18 (0.03) | 0.21 (0.03) | 0.18 (0.02) |
| Plastid C1 class II fructose bisphosphate aldolase | 0.30 (0.05) | 0.32 (0.04) | 0.27 (0.04) | 0.38 (0.02) | 0.33 (0.03) | 0.32 (0.03) |
| RecName: Full=Peridinin chlorophyll a-binding protein, chloroplastic; Short=PCP; Flags: Precursor | 0.33 (0.05) | 0.26 (0.03) | 0.26 (0.02) | 0.25 (0.06) | 0.27 (0.04) | 0.31 (0.02) |
| RecName: Full=Peridinin chlorophyll a-binding protein, chloroplastic; Short=PCP; Flags: Precursor | 0.57 (0.04) | 0.60 (0.08) | 0.45 (0.12) | 0.55 (0.03) | 0.49 (0.07) | 0.44 (0.08) |
| RecName: Full=Peridinin chlorophyll a-binding protein, chloroplastic; Short=PCP; Flags: Precursor | 0.49 (0.05) | 0.62 (0.07) | 0.79 (0.08) | 0.39 (0.11) | 0.57 (0.13) | 0.67 (0.10) |
| RecName: Full=Peridinin chlorophyll a-binding protein, chloroplastic; Short=PCP; Flags: Precursor | 0.64 (0.04) | 0.59 (0.02) | 0.55 (0.07) | 0.58 (0.06) | 0.58 (0.01) | 0.70 (0.04) |
| RecName: Full=Peridinin chlorophyll a-binding protein, chloroplastic; Short=PCP; Flags: Precursor | 0.97 (0.09) | 0.56 (0.08) | 0.91 (0.10) | 0.67 (0.29) | 0.68 (0.07) | 0.85 (0.08) |
| RecName: Full=ZP domain containing protein; Flags: Precursor | 11.97 (0.71) | 10.4 (0.67) | 10.88 (0.49) | 12.28 (0.26) | 11.67 (0.37) | 11.1 (0.72) |
| RecName: Full=ZP domain containing protein; Flags: Precursor | 6.22 (0.57) | 6.43 (0.59) | 7.96 (1.15) | 7.15 (0.87) | 6.21 (0.41) | 8.74 (1.00) |
| Ribonuclease Z | 0.12 (0.02) | 0.09 (0.01) | 0.13 (0.03) | 0.11 (0.03) | 0.09 (0.01) | 0.08 (0.02) |
| Ribulose 1,5-bisphosphate carboxylase oxygenase large subunit precursor | 0.22 (0.04) | 0.27 (0.07) | 0.36 (0.14) | 0.20 (0.05) | 0.14 (0.02) | 0.13 (0.06) |
| Ribulose 1,5-bisphosphate carboxylase oxygenase large subunit precursor | 0.34 (0.02) | 0.27 (0.02) | 0.25 (0.06) | 0.27 (0.01) | 0.20 (0.01) | 0.27 (0.04) |
| Ribulose 1,5-bisphosphate carboxylase oxygenase large subunit precursor | 0.95 (0.05) | 1.18 (0.10) | 1.14 (0.16) | 0.94 (0.16) | 1.02 (0.10) | 0.99 (0.17) |
| Triosephosphate isomerase | 0.11 (0.01) | 0.14 (0.02) | 0.09 (0.01) | 0.14 (0.03) | 0.11 (0.01) | 0.14 (0.00) |
| **Symbiont Proteins That Show Significant Changes in Abundance** | | | | | | |
|  | **18:40** | **22:40** | **02:40** | **6:40** | **10:40** | **14:40** |
| Chloroplast light harvesting complex protein | 0.05 (0.01) | 0.04 (0.01) | 0.02 (0.00) | 0.06 (0.02) | 0.06 (0.01) | 0.04 (0.01) |
| Chloroplast light harvesting complex protein | 0.44 (0.06) | 0.41 (0.06) | 0.33 (0.05) | 0.31 (0.05) | 0.49 (0.03) | 0.22 (0.03) |
| Elongation factor Tu (chloroplast) | 0.25 (0.02) | 0.28 (0.01) | 0.21 (0.03) | 0.31 (0.03) | 0.27 (0.02) | 0.31 (0.02) |
| Enoyl-acp reductase | 0.22 (0.07) | 0.07 (0.01) | 0.19 (0.03) | 0.21 (0.02) | 0.18 (0.02) | 0.24 (0.03) |
| Glutamate semialdehyde synthase | 0.17 (0.06) | 0.03 (0.00) | 0.15 (0.02) | 0.18 (0.02) | 0.14 (0.01) | 0.18 (0.01) |
| Mitochondrial 60 kDa heat shock | 0.19 (0.03) | 0.15 (0.04) | 0.35 (0.02) | 0.12 (0.06) | 0.13 (0.05) | 0.12 (0.05) |
| RecName: Full=ZP domaincontaining protein; Flags: Precursor | 0.08 (0.01) | 0.07 (0.02) | 0.08 (0.01) | 0.06 (0.03) | 0.08 (0.01) | 0.02 (0.01) |
| Ribulose 1,5-bisphosphate carboxylase oxygenase large subunit precursor | 0.30 (0.02) | 0.16 (0.02) | 0.18 (0.04) | 0.19 (0.03) | 0.16 (0.01) | 0.18 (0.04) |
| Ribulose 1,5-bisphosphate carboxylase oxygenase large subunit precursor | 0.29 (0.02) | 0.30 (0.04) | 0.26 (0.03) | 0.33 (0.04) | 0.36 (0.02) | 0.39 (0.03) |
